# Gender difference in components of metabolic syndrome among patients of type 2 diabetes

**DOI:** 10.12669/pjms.314.6714

**Published:** 2015

**Authors:** Muhammad Ahmad Alamgir, Riaz Ahmad Javid, Abdul Hameed, Irum Mustafa

**Affiliations:** 1 Dr. Muhammad Ahmad Alamgir, MBBS, MCPS, FCPS, DPH, Assistant Professor of Medicine, BVH/QAMC, Bahawalpur, Pakistan; 2 Dr. Riaz Ahmad Javid, MBBS, FCPS, Senior Registrar Medicine, BVH, Bahawalpur, Pakistan; 3 Dr. Abdul Hameed, MBBS, FCPS Assistant Professor of Medicine, Multan Medical & Dental College, Multan, Pakistan; 4 Dr. Irum Mustafa, Demonstrator, Quaid-e-Azam Medical College, Bahawalpur, Pakistan

**Keywords:** Diabetes mellitus, Gender, Metabolic syndrome

## Abstract

**Background and Objectives:**

Diabetes mellitus, hypertension, obesity and dyslipidemia are the integral components that constitute metabolic syndrome. It has emerged as cause of substantial mortality with gender difference. To compare the gender characteristics of metabolic syndrome in subjects of type 2 diabetes mellitus.

**Methods:**

This observational comparative prospective study was conducted in medical ward of BVH Bahawalpur over period of 6 month from January 2014 to June 2014. Sample size of 100 diagnosed cases of type 2 diabetics (comprising equal number of males and females) were collected. As defined by WHO and IDF, variables of metabolic syndrome studied were BMI, hypertension, hyperglycemia and hyperlipidemia. The lipid values were interpreted in normal or high risk category by applying NCEP ATPIII criterion. Fasting sample were collected for sugar and lipid profile. Anthropometric, demographic and biochemical data was tabulated. Continuous and categorical variables were displayed as mean ± standard deviation and percentage respectively. Student ‘t’ test was use to assess the difference for the lipid profile at 5% significance level.

**Results:**

Female patients were found to be overweight, hypertensive and had uncontrolled hyperglycemia as compared to male subjects. Their systolic blood pressure was 150±25.75 and diastolic was 90±17mm/hg. The mean blood glucose concentration was 246.64±105mg/dl. In age group 35-44 years, the mean triglycerides and cholesterol levels in females were 184.54±42.05 and 192.5±34mg/dl respectively. HDL-C was 33.2±5.19mg/dl. In females with age group of 45-54 years, the mean concentration of triglycerides, total cholesterol and HDL-C were 217.75±77.6mg/dl, 190.95±14.4mg/dl and 31.75±3.8mg/dl respectively, and all were in high risk category when compared to cut off values determined by NCEP/ADA. While in females of age group 55-64 years, the values of TGs, total cholesterol and HDL-C were 204±154.11mg/dl, 200.09± 47.8 mg/dl and 33.4±4.6mg/dl respectively and again all these values were significantly raised.

**Conclusion:**

Female genders were affected in all respects. They had higher BMI, hypertension, hyperglycemia and hyperlipidemia as compared to male counterparts.

## INTRODUCTION

Diabetes mellitus is increasingly considered as a pandemic worldwide. It is recently estimated that more than 180 million people are affected with diabetes globally and number is likely to hit more than double by the year 2025.^1^ Pakistan ranks 7^th^ on this prevalence list with 7 million diabetic populations.^2^

Almost 80% of death in diabetic patients occurs due to stroke and coronary heart diseases. Uncontrolled hyperglycemia in addition to other risk factors like obesity, dyslipidemia and hypertension is associated with large decrease in life expectancy.^3^,^4^

Studies consistently demonstrate that just optimal glycemic control do not reduce the complications.^5^ The diabetics commonly have association of hypertension, obesity and dyslipidemia; the cluster of risk factors termed as metabolic syndrome.^6^,^7^ These risk factors act synergistically for development of atherosclerosis. It necessitates aggressive approach for management of these metabolic risk factors to prevent the incidence of cardiovascular complications.

The epidemiological research trials show that there is gender disparity among these components of metabolic syndrome. The estrogen affect glucose and lipoprotein metabolism. Literature suggests that women with diabetes are more likely to have uncontrolled hyperglycemia, hypertension and dyslipidemia as compared to male counterparts.^8^-^10^ So they experience worse cardiovascular events. On the other hand, some case control studies have generally failed to show this relationship and have conflicting results.

Although a lot of work has been done on diabetes but the concept behind this article arises from the fact that females are neglected section of society particularly in South Punjab. The present study seeks to compare the variables of metabolic syndrome like blood pressure, lipid profile and glycemic status among diabetic males and females and to assess if these levels are normal or differ with cut off values already known internationally by ADA and NCEP.

This study aimed to explore and compare the individual components of metabolic syndrome among male and female type 2 diabetics.

## METHODS

This comparative and prospective observational study was conducted from January 2014 to June 2014. Hundred diagnosed type 2 diabetics (comprising equal number of males and females), between age of 25-65 years were randomly selected for study. History and clinical examination was recorded on each Performa after taking a formal consent. There was no difference in duration of diabetes or type of treatment.

### Exclusion criterion

Patients with history of hypothyroidism, nephrotic syndrome and those taking steroids, oral contraceptive pills or lipid lowering drugs, were excluded from study.

### Measurements

Blood pressure was measured twice while patients were sitting for 05 minutes. According to ADA criteria, the diabetic patients having blood pressure >140/90mm / hg were considered as hypertensive.

### BMI

It was measured according to formula of weight in Kg divided by the square of height in meters: Classifying overweight (BMI 25-29) and obese (BMI>30).

### Working definition of metabolic syndrome for the study

Diagnosis of metabolic syndrome was determined by using NCEP ATP III / ADA criterion as it is internationally agreed upon for diabetics.^11^,^12^ It is defined as sum of three or more of following with their cut off values; overweight (BMI>25), hypertension (BP>140/90 mm/hg), triglycerides > 150 mg / dl, HDL-C< 35 mg for men and less than 45 mg / dl for women and total cholesterol > 190 mg / dl. Higher the HDL-C level, beneficial it is because it is considered as good cholesterol.

Blood glucose was determined by enzymatic colour test on spectrophotometer. Fasting lipid levels were taken centrifuged within 15-minutes and their values were determined by ELIZA (Rendox Labs). Lipid parameters like total cholesterol, triglycerides, and HDL-C were recorded and these collected data were fed and analyzed through computer software SPSS version 11. Mean value of quantitative data were represented as mean SD. Student ‘t’ test was applied for lipid levels, values were tested at 5% significance level and the P value <0.05 was considered as significant.

## RESULTS

This study endeavors to provide the comparative picture of demographic and metabolic parameters among male and female diabetics. Table-I shows gender distribution of subjects among various age matched. It shows that maximum patients present in middle and old age groups. Mean total duration of diabetes was 5.69±4.24 years. A comparison of demographic and glycemic status among male and female subjects is mentioned in Table-II. It shows that BMI, blood pressure, glycemic status was significantly higher in female gender. This table also shows characteristic parameters. The systolic blood pressure showed a significant difference in both sexes. It was 135.71±21.29 in males and 150±25.72 in females, while diastolic blood pressure was 80.21±13.38 and 90.6±17.60 respectively. The mean blood glucose in males was 208.57± 101.6 mg% and 246.64±105.06 mg% in females, showing a significant difference. Calculated BMI was 26.01±3.92 in males and 29.21±5.58 in females groups.

**Table-I:** Age and sex distribution among patients studied.

Age group	Males	Percentage	Females	Percentage
25-34	14	28%	6	12%
35-44	10	20%	12	24%
45-54	10	20%	10	20%
55-64	16	32%	22	44%
Total	50		50	

**Table-II:** Comparison of demographic parameters between male & female subjects.

Variables	Units	Males	Females
Systolic B.P	mm/hg	135.71±21.29	150±25.72
Diastolic B.P	mm/hg	80.21±13.38	90±17.60
Blood Glucose	mg/dl	208.57±101.6	246.64±105.06
BMI	Normal < 25	26.01±3.92	29.21±5.58

Table-III shows the mean values of serum triglycerides (TGs), total cholesterol and high density lipoproteins (HDL-C) in males and females of various age groups and tabulated accordingly. It is noteworthy that their values were raised in females as compared to male subjects. In the age group 25-34 years, the mean triglycerides concentrations in male and female groups was 167.66±54.3 and 181.6±40.5 mg/dl (P-value = 0.7035), the total cholesterol was 184.33±11.94 and 195.4±30.5 mg/dl (P-value=0.4093) and HDL-C was 34.5±2.5 and 32.6±4.3 mg/dl (P=0.3932) respectively.

**Table-III:** Mean concentration of triglycerides, total cholesterol and HDL-C among various age matched.

Age Group	Triglycerides		P-Value	Total Cholesterol		P-Value	HDL-C		P-Value
	M	F		M	F		M	F	
25-34	167.66±54.3	181.6±40.5	0.7035	184.33±11.94	195.4±30.5	0.4093	34.5±2.5	32.6±4.3	0.3932
35-44	162.67±57.2	184.5±42.05	0.4837	187.5±62.5	192.5±34.3	0.8696	35.25±8.4	33.2±5.19	0.6308
45-54	159.3±21.93	217.75±77.6	0.1437	167.6±28.59	190.75±14.4	0.1445	39±2.94	31.75±3.8	0.0097
55-64	173.38±61.8	204±154.11	0.6039	176.86±51.42	200.09±47.8	0.3250	34.36±7.58	33.4±4.6	0.7352

For the age group of 35-45 years the values of TGs in men and women were 162.67±57.2 and 184.5±42.05 mg/dl (P=0.4837), the total cholesterol was 187.5±62.5 and 192.5±34.5 mg/dl. (P=0.8616) and HDL-C was 34.5±2.5 and 32.6±4.3 mg/dl (P=0.6308) respectively.

In the age group of 45-54 years the mean concentration of TGs in males and female groups were 159.3±21.93 and 217.75±77.6 mg/dl (P=0.1437), the total cholesterol was 167.6±28.54 and 190.75±14.40 mg/dl (P=0.1445) in both genders respectively and HDL-C was 39±2.94 and 31.75±3.8mg/dl (P=0.0097) respectively.

So collectively the lipid levels were significantly raised as compared to male subjects in middle age groups. Similar paradigm was observed in age group of 55 – 64 years. In this age group the values of TGs in male and female subjects were 173.38±61.8 and 204±154.11 mg/dl (P=0.6039), the cholesterol concentration was 176.88±5.42 and 200.09±47.8 (P=0.3250) and HDL-C was 34.369±7.58 and 33.4±4.6 mg/dl (P=0.7352) respectively.

## DISCUSSION

There is a concept that pathogenesis of cardio-metabolic risk factors differ between diabetic men and women. The observation of sex difference in body fat distribution, insulin resistance, sex hormones (estrogen and progesterone) and effect of glucose further support this hypothesis. Spectacular advances in evidence based medicine have shown that diabetic middle aged women are 8 times more likely to develop cardiovascular events than non-diabetic.^9^,^10^,^12^

The present study was designed to compare the core component of metabolic syndrome like obesity, hypertension, dyslipidemia (hypercholesterolemia, decrease HDL-C, increased triglycerides) among diabetic males and females. Our results had paramount importance. As far as the anthropometric parameters are concerned, our female population group was found to be obese as compared to male group. It is advantageous here to compare with recent database information reported by international organization. According to large population survey conducted by ADA/AHA, female diabetics were more obese as compared to male diabetics (13% and 10% respectively).^13^,^14^

In addition to obesity, our female group also had hypertension and uncontrolled hyperglycemia. These results are generally in accordance to many other studies. Results of a large population survey conducted by Shera AS et al., are important. According to their study, 79% of diabetic females and 20% of males in Pakistan were overweight. Similarly more than 70% females had uncontrolled hyperglycemia and found to be hypertensive as well.^15^ Habib S et al.,^16^ has reported similar findings. Their female group was more obese than males. They were hypertensive and high level of total cholesterol and triglycerides were observed respectively.

As far as the pattern of lipoproteins is concerned, it is high time to compare with the international cut off values in diabetics as determined by NCEP ATP III and ADA.^12^,^13^ According to their position statement, HDL-C (good cholesterol) should be essentially more than 45mg/dl for diabetic women and more than 35mg/dl in diabetic men. Total cholesterol should be below 160mg/dl in diabetics as lower the cholesterol beneficial it is. Moreover TGs should be. So our female cohort had significantly elevated level of total cholesterol and triglycerides along with low HDL-C. An important breakthrough in our results was noted that lipid abnormalities increase with age in females thus posing a potential high cardiovascular risk. So collectively all the components of metabolic syndrome including (hypertension, hyperglycemia and dyslipidemia) were found in our female group.

Cluster of these risk factors was also shown by Mohsin A and Zafar J et al.^17^ Their females were affected more than males in all respects. Of 106 patients, 91 had metabolic syndrome and majority (95%) were females. Low HDL-C was present in all females. 78% females had elevated TGs as well. Their 50% female had all components of metabolic syndrome except hypertension and difference was statistically significant. In a local study conducted by Nazeer M et al., and Alamgir et al., in his thesis, had also shown these risk factors in females and particularly in postmenopausal age.^18^ Their patients had uncontrolled hyperglycemia (odd ratio 2.66 CI 1.3- 5.1) hyperlipidemia (odd ratio 2.25 CI 1.2-2.3) and increased weight (odd ratio 2.16 CI 1.1-4.2) reached statistical significance. These findings bear important public health implications and demands urgent consideration.

Most of the studies had shown positive relationship while a few led to controversial results as well. It depends of ethnic groups and nations. Dyslipidemia showed statistically weak relationship in some Indian and African women while it had modest or strong association in native postmenopausal Pakistani population. In the Indian research, Kumar SV et al., has shown contrary results. Their male and female subjects had equal level of triglycerides (186mg% each), HDL-C (22mg% each) and blood pressure was 130/80mm/hg in both subjects.^19^ In the analysis of an international research held by Ogbera AO,^20^ the frequency of occurrence of all components were similar to both groups and no statistically significant difference was noted. The possible explanation is that in our work local hospital population was examined while international trials have different geographical variations.

Most of the above reported studies have shown clinical interest leading to appraisal that diabetic females are affected in all respects. Limitation of our study was small sample size and based upon single ward data. It is suggested that further longitudinal studies may be conducted in community to validate these findings.

## CONCLUSION

The cardio metabolic risk factors are significantly associated with female diabetics as compared to male counter parts. The healthcare providers should focus on holistic and integral approach to the patients for lifestyle modifications, exercise and heart healthy diet. Hope is the engine of soul. These preventive measures along with lipid lowering drugs are essential to help combat the incidence of cardiovascular complications.

## References

[1] American Diabetic Association (2013). Fast Facts. Data and Statistics about diabetes. http://www.diabetes.org/diebeties-basic-staticsstatostocs.

[2] Shera AS, Basit A, Fawad A, Hakim R (2010). Pakistan National Diabetic Research Survey. Primary Care Diabetes.

[3] Anderson TJ, Grégoire J, Hegele RA, Couture P, Mancini GB, McPherson R (2013). 2012 update of Canadian Cardiovascular Society. Guidelines for prevention of CV events in adults. Can J Cardiol.

[4] Bell DSH (2011). Impact of dyslipidemia in diabetes and obesity.

[5] Gushman VC, Accord Study Group (2010). Effect of intensive glycemic control in type 2-D.M. N Eng J Med.

[6] Bayturan O, Tuzcu EM, Lavoie A, Hu T, Wolski K, Schoenhagen P (2010). The metabolic syndrome, its component risk and progression of CHD. Arch Intern Med.

[7] Simmons RK, Alberti KG, Gale EA, Colagiuri S, Tuomilehto J, Qiao Q (2010). Metabolic syndrome. A useful concept or clinical tool. Report of WHO expert consultation. Diabetologica.

[8] Gobl CS, Brannath W, Bozkurt L, Handisurya A, Anderwald C, Luger A (2010). Sex specific difference in glycemic control and CV risk factors in elderly diabetic. Gend Med.

[9] Mascarenhas-Melo F, Marado D, Palavra F, Sereno J, Coelho Á, Pinto R (2013). Diabetes abrogates Sex difference and aggravates CV risk in post-menopausal women. CV Diabetol.

[10] Yu MK, Courtney Rees Lyles CR, Bent-Shaw LA, Young BA (2013). Sex Disparities in Diabetes Process of Care Measures and Self-Care in High-Risk Patients. J Diabetes Res.

[11] Ahmad A (2012). Metabolic syndrome in type 2 diabetes. Comparison of WHO, modified ATP III and IDF criteria. J Pak Med Assoc.

[12] Mosca L, Benjamin EJ, Berra K, Besancon JL, Dolor RJ, Lloyd-Jones DM (2011). Guidelines for prevention of CHD in women –2011 update. American Diabetic Association. Circulation.

[13] Grundy M (2014). American Diabetic Association-Scientific Statement. Diabetes with CHD. Circulation.

[14] (2009). US Preventive services Task Force. Using nontraditional risk factors, USPSTF recommendations. Ann Intern Med.

[15] Shera AS, Jawad F, Maqsood A (2007). Prevalence of diabetes in Pakistan. Diabetes Res Clin Pract.

[16] Habib SS (2013). Gender difference in lipids and glycemic control in patients with type 2 diabetes. Rawal Med J.

[17] Mohsin A, Zafar J, Nisar YB, Imran SM (2007). Frequency of metabolic syndrome in adult type 2 diabetic presenting to PIMS. J Pak Med Assoc.

[18] Naseer M, Naveed T, Amanullah (2010). A case control study of risk factors for coronary artery disease in Pakistani females. Ann KEMU.

[19] Kumar SV, Nagesh A, Leena M, Shravani G (2013). Incidence of metabolic syndrome and its characteristics of patients attending diabetic out patients clinic. J Nat Sc Biol Med.

[20] Ogbera AO (2010). Prevalence and gender distribution of metabolic syndrome. Diabetology and metabolic syndrome.

